# The US population-level burden of cow's milk allergy

**DOI:** 10.1016/j.waojou.2022.100644

**Published:** 2022-04-21

**Authors:** Christopher M. Warren, Avni Agrawal, Divya Gandhi, Ruchi S. Gupta

**Affiliations:** a Center for Food Allergy and Asthma Research, Northwestern University Feinberg School of Medicine, 750 N Lake Shore Drive, 6^th^ Floor, Chicago, IL, 60611, USA; b College of Osteopathic Medicine –LECOM Bradenton, 5000 Lakewood Ranch Blvd, Bradenton, FL, 34211, USA

**Keywords:** Cow's milk allergy, Food allergy, Food hypersensitivity, Psychosocial burden, Epidemiology

## Abstract

**Background:**

Cow's milk is a food allergen of public health importance both in the United States and globally. Its natural history and epidemiology have been most studied among infants and young children, but the public health burden it places on adults and older pediatric populations remains unclear. This study comprehensively characterizes the US population-level burden of cow's milk allergy (CMA), including its prevalence, severity, health care utilization, psychosocial impact, natural history, and other associated factors—including ability to tolerate extensively heated milk products—within a large, nationally-representative survey sample of US households.

**Methods:**

A cross-sectional survey was administered via web and telephone. Population level inference was based on data collected from participants recruited from National Opinion Research Center (NORC) at the University of Chicago's probability-based *AmeriSpeak* panel. Established dual-sample complex survey calibration methods were used to augment this sample with additional participants recruited through Survey Sampling International to increase the precision of the resulting estimates. In total, surveys were administered to a nationally representative sample of 51 819 US households from October 1, 2015, through September 31, 2016. Self-report responses from 40 453 adults and parent-proxy report for 38 408 children were analyzed.

**Results:**

Analyzing survey responses from 78 851 individuals, an estimated 4.7% (95% CI, 4.4%–4.9%) of the US population reported current CMA, whereas 1.9% (95% CI, 1.8%–2.1%) met symptom-report criteria for convincing IgE-mediated allergy. An estimated 0.9% (95% CI, 0.8%–1.0%) had CMA that met convincing symptom-report criteria and was physician diagnosed. Female and White respondents were more likely to report outgrowing CMA relative to males and those reporting other races and ethnicities. Individuals with CMA who reported tolerance to baked milk products were less likely to report severe reaction histories (33.5% vs 42.7%; p = .03), a lifetime history of food allergy-related emergency department visits (43.4% vs. 55.8%; p = .005), and treating a milk-allergic reaction with epinephrine (10.6 vs. 18.9%; p = .003). These individuals also exhibited less psychosocial burden on the validated Food Allergy Independent Measure (FAIM) than their counterparts with CMA who were allergic to baked forms of milk.

**Conclusion:**

These data indicate a discrepancy in reported rates of allergy to cow's milk among the US general population ranging from approximately 1 in 20 with reported CMA to fewer than 1 in 50 with physician-confirmed CMA. However, they suggest a substantial population-level burden of CMA, including substantial healthcare utilization, psychosocial burden and nutritional impacts—particularly among the approximately 30% of individuals with CMA who cannot tolerate baked forms of milk.

## Introduction

Food plays an integral role in our daily lives. The ubiquitous nature of food is taxing for individuals with food allergy who must avoid specific food allergens in the many environments where they can appear. Previous studies have shown that IgE-mediated food allergy affects 8% of children and 10%^1^ of adults in the United States, causing significant impacts on both physical and psychological health.^2^ Food allergies also cause significant economic impacts; in the pediatric population alone, food allergy has been estimated to cost an estimated $24.8 billion each year in the United States ($4184 per year per child), imposing an unknown, but likely substantial, financial burden on affected adults as well as the families of those individuals.^3^

Previous epidemiological data indicate that cow's milk is one of the most common allergens, particularly among infants and young children. While frequently outgrown, cow's milk remains one of the most common food allergens among school-age children and adolescents. However, to date, its impact on adult populations in the United States and globally remains largely unknown. Data indicate that individuals with cow's milk allergy (CMA) have a lower health-related quality of life (QoL), not only compared to the general population, but even among those with other common allergies such as peanut allergy—a more frequently accommodated and, perhaps, easily avoidable allergen.^4^ Furthermore, numerous studies suggest that the cow's milk avoidance diet required for management of IgE-mediated CMA may have deleterious effects on growth and nutrition outcomes—highlighting the importance of accurate diagnosis and effective clinical management of CMA.

Current literature estimates that CMA affects 2–3% of US infants, rendering it the most prevalent allergen in this population.^4^ However, the prevalence of CMA over the lifecourse remains largely unknown. A large cohort study conducted in Israel found that 57% of participants outgrew their CMA within 4–5 years and a majority outgrew their allergy by age 2.^5^ Interestingly, a recent epidemiologic study found that CMA is a problem of growing global importance with significant lifelong effects.^6^

Reactions to cow's milk can be divided into immunoglobulin E (IgE)-mediated and non-IgE-mediated responses. An IgE-mediated response to cow's milk is defined as a type I hypersensitivity reaction to cow's milk proteins, which shows a symptomatic reaction within seconds to minutes of ingestion. While the amount of reported IgE-mediated CMA cases remains high, misdiagnosis is common. The non-IgE mediated immune reactions include enzyme deficiencies such as lactose malabsorption and intolerance. Due to some shared symptomatology and the fact that both are elicited by consumption of cow's milk-containing products, it is believed that many individuals with non-IgE-mediated reactions mistakenly believe themselves to have IgE-mediated CMA and therefore engage in strict avoidance.

Adding complexity, cow's milk-induced reactions can vary in severity based on the type of milk product consumed, such as baked goods that contain extensively heated forms of milk and/or the other food matrix components present within the milk product which may impact IgE binding or other relevant physicochemical properties.^7^ Studies suggest that among clinical samples from individuals diagnosed with CMA, a majority can tolerate baked forms of milk.^8^ Furthermore, data from small, selected samples suggest that reactivity to baked milk may be associated with a more severe milk allergy phenotype.^9^ However, in regards to management of CMA within the general milk-allergic population, little is known regarding possession and/or use of epinephrine as well as the extent to which reactions to cow's milk results in emergency room visits or other severe allergic manifestations. This study aimed to leverage data from a large, nationally representative survey sample of US households to characterize the current population-level burden of CMA across the lifespan, while also characterizing clinical and phenotypic differences between individuals with CMA who can and cannot tolerate baked forms of milk.

## Methods

A national cross-sectional food allergy questionnaire was administered via web and telephone from October 1, 2015 through September 31, 2016. The institutional review boards (IRB) of the participating research sites approved the study protocol and activities. Either written or oral informed consent was obtained from all study participants. Study participants were first recruited from the probability-based *AmeriSpeak* panel, with 7218 responses of 14 095 invitees resulting in a survey completion rate of 51.2%. Each child and adult participant were assigned a base, study-specific sampling weight equal to their or their responding parent's nonresponse-adjusted *AmeriSpeak* sampling weight. Iterative proportional fitting methods were applied to rake adult sampling weights to external population totals associated with age, sex, educational attainment, race/ethnicity, housing tenure, telephone status, and Census Division to improve external validity. Child-specific weights were further adjusted to account for random selection of as many as 3 children per household and raked to external pediatric US population totals. To increase precision and ensure sufficient sample size among key subpopulations, prevalence estimates gleaned from population-weighted *AmeriSpeak* responses were augmented by calibration-weighted, nonprobability-based responses obtained through Survey Sampling International. The final, combined sample weight was derived by applying an optimal composition factor that minimizes mean square error associated with food allergy prevalence estimates. Detailed information regarding the complex survey sampling, weighting, and analysis methods used was previously published.^1^ In total, surveys were completed by 51 819 US households from all 50 states and the District of Columbia, providing parent-proxy responses for 38 408 children and self-report for 40 443 adults.

The primary outcome measure for the present study was the prevalence of cow's milk allergy. Reported cow's milk allergies were considered to be convincing if the most severe reaction reported to that food included at least one symptom on the stringent symptom list developed by our expert panel (Supplemental Figure 1), even if such allergies were reported to be physician-diagnosed. Convincing cow's milk allergies for which parents reported a doctor's diagnosis were considered physician-confirmed. A summary of how these case definitions were applied is provided in Supplemental Figure 2. A severe reaction history to cow's milk was indicated by parent-report of multiple specific stringent symptoms occurring within 2 or more of the following 4 organ systems (skin/oral mucosa, gastrointestinal, cardiovascular, and respiratory) in response to the question “Think back to the most severe allergic reaction to milk that your child has ever had. What were [his/her/their] symptoms?” Both current and outgrown milk allergies were assessed, as was the ability to tolerate baked milk. Complete descriptions of the survey development, testing, categorization of allergy type have been published previously.^10^ Briefly, CMA onset was determined by asking respondents to report their age/their child's age at the time of their first allergic reaction to cow's milk (in months if onset occurred during the first year of life and in years for onset after age 1). “Outgrown allergy” was assessed by asking respondents “Have you/your child ever outgrown any food allergies? (ie, Have you/your child ever been allergic to a food that you/he/she can now eat without having an allergic reaction?)”. This question was followed-up with “Which food allergies have you/your child outgrown?” (Do not include foods that you/your child still cannot eat due to a current allergy), along with a list of all specific foods to which respondents reported ever having an allergic reaction. Finally, specific allergic reaction symptoms and history of physician-diagnosis were queried and reaction symptomatology was classified as convincingly IgE-mediated or not in an identical fashion as for current allergies. Only reportedly outgrown allergies meeting the stringent symptom-report criteria described in the supplemental figures were considered as such in the present manuscript.

To assess the psychosocial burden of living with a food allergy, the food allergy independent measure (FAIM) was administered to all respondents reporting a current food allergy. This validated measure is comprised of 6 questions, which are scored on a 1-to-7-point scale with higher scores indicative of greater psychosocial burden and lower food allergy-related quality of life (QoL). FAIM scores for the parent-proxy child response form (<13 years), parent-proxy teen form (13–17 years), and adult^11^ self-report forms (18+ years) were calculated as described^12^ by the scale authors. Similarly, height and weight at the time of survey administration were obtained via parent-proxy or self-report, and body mass index (BMI) and weight categories were calculated using standardized methods based on the World Health Organization definitions.^13^

### Statistical analysis

Complex survey-weighted means and proportions were calculated to estimate US population prevalence of CMA and other characteristics using STATA 16 svy: prefix. Multiple linear regression models were fit to estimate covariate-adjusted effects of current CMA on reported body mass indices, which were examined for goodness of fit with the data via diagnostic plots of residuals against fitted values and quantile-quantile plots. Cluster robust standard errors accounted for clustering of multiple individuals within the same household (eg, adult self-report and child parent-proxy report). The FAIM exhibited excellent internal consistency (Cronbach's α > 0.8), and confirmatory factor analysis of the FAIM concluded that a two-factor solution exhibited excellent fit^12^ to the data (Confirmatory Fit Index [CFI]≥0.950; Root Mean Square Error of Approximation [RMSEA]<0.08). Two-sided hypothesis tests with a two-sided P < .05 were used to indicate statistical significance.

## Results

Of the 51 819 US households surveyed, responses for 40 443 adults and 38 408 children were included. As shown in Table 1, the demographic characteristics of the weighted sample are representative of the general US population.

**Table 1:** Distribution of weighted sample demographics and comorbid allergic disease.

Variable	US Population % (95% CI)	With convincing milk allergy % (95% CI)	With other allergies^a^ % (95% CI)
Race and ethnicity			
Asian, non-Hispanic	3.7 (3.5–4.0)	2.9 (2.2–3.8)	4.8 (4.0–5.7)
Black, non-Hispanic	12.0 (11.6–12.5)	14.3 (11.8–17.4)	13.3 (12.0–14.8)
White, non-Hispanic	62.2 (61.4–62.9)	52.9 (49.0–56.7)	58.2 (56.1–60.3)
Hispanic	17.4 (16.7–18.1)	22.5 (19.0–26.5)	18.5 (16.8–20.3)
Multiple and other	4.7 (4.4–4.9)	7.4 (5.4–10.1)	5.2 (4.3–6.3)
Born in the US			
Yes	93.0 (92.6–93.3)	94 (92.0–95.6)	92.8 (91.5–94.0)
No	7.1 (6.7–7.4)	6.0 (4.4–8.1)	7.2 (6.0–8.5)
Sex			
Female	51.1 (50.5–51.6)	63.7 (59.9–67.2)	59.9 (57.8–61.9)
Male	48.9 (48.4–49.5)	36.3 (32.8–40.2)	40.1 (38.1–42.2)
Age			
<1 year	1.2 (1.1–1.3)	0.9 (0.6–1.4)	0.22 (0.15–0.33)
1 year	1.1 (1.0–1.2)	1.9 (1.2–3.0)	0.89 (0.59–1.34)
2 years	1.3 (1.2–1.4)	2.9 (1.5–5.6)	0.89 (0.66–1.21)
3–5 years	3.6 (3.5–3.8)	5.3 (3.9–7.1)	2.4 (2.0–3.0)
6–10 years	6.2 (6.0–6.5)	6.3 (4.9–8.1)	5.6 (4.9–6.3)
11–13 years	3.6 (3.5–3.9)	2.2 (1.6–3.0)	3.7 (3.0–4.4)
14–17 years	5.2 (5.0–5.5)	3.1 (2.4–3.9)	4.3 (3.6–5.0)
18–29 years	16.7 (16.2–17.2)	20.7 (17.7–24.2)	19.3 (17.6–21.1)
30–39 years	13.2 (12.8–13.5)	15.8 (13.4–18.6)	18.0 (16.3–19.7)
40–49 years	13.0 (12.6–13.4)	13.2 (10.9–15.9)	12.5 (11.2–13.8)
50–59 years	14.0 (13.6–14.4)	13.6 (11.4–16.1)	16.3 (14.7–17.9)
60+ years	20.8 (20.3–21.3)	14.1 (11.8–16.8)	16.1 (14.5–17.8)
Annual household income, $(USD)			
<25,000	16.5 (16.0–17.0)	16.7 (14.2–19.4)	15.0 (13.5–16.6)
25,000–49,000	22.0 (21.4–22.6)	22.8 (20.0–25.8)	21.8 (20.2–23.5)
50,000–99,999	31.0 (30.3–31.7)	34.2 (30.8–37.7)	34.0 (32.1–36.1)
100,000–149,000	19.5 (18.9–20.2)	17.2 (13.9–21.0)	21.0 (19.1–23.0)
≥150,000	11.0 (10.5–11.6)	9.2 (7.0–12.1)	8.2 (7.1–9.5)
Other conditions			
Asthma	12.2 (11.8–12.7)	27.0 (23.6–30.7)	26.1 (24.3–27.9)
Eczema	6.5 (6.2–6.9)	15.7 (13.4–18.3)	12.2 (10.8–13.7)
Environmental allergies	19.5 (19.0–20.0)	33.1 (29.9–36.5)	32.5 (30.5–34.5)
EoE	0.17 (0.14–0.22)	0.81 (0.47–1.39)	0.6 (0.4–0.9)
BMI [Mean (95% CI)]	22.6 (22.5–22.8)	21.0 (20.2–21.9)	22.8 (22.3–23.2)

Note: These are weighted proportions.

Abbreviations: BMI, body mass index; CI, confidence interval; EoE, eosinophilic esophagitis.

a The other top 8 food allergies: peanut, tree nut, egg, wheat, soy, fish, shellfish, sesame

### CMA prevalence by demographic and clinical atopic characteristics

**Fig. 1:**
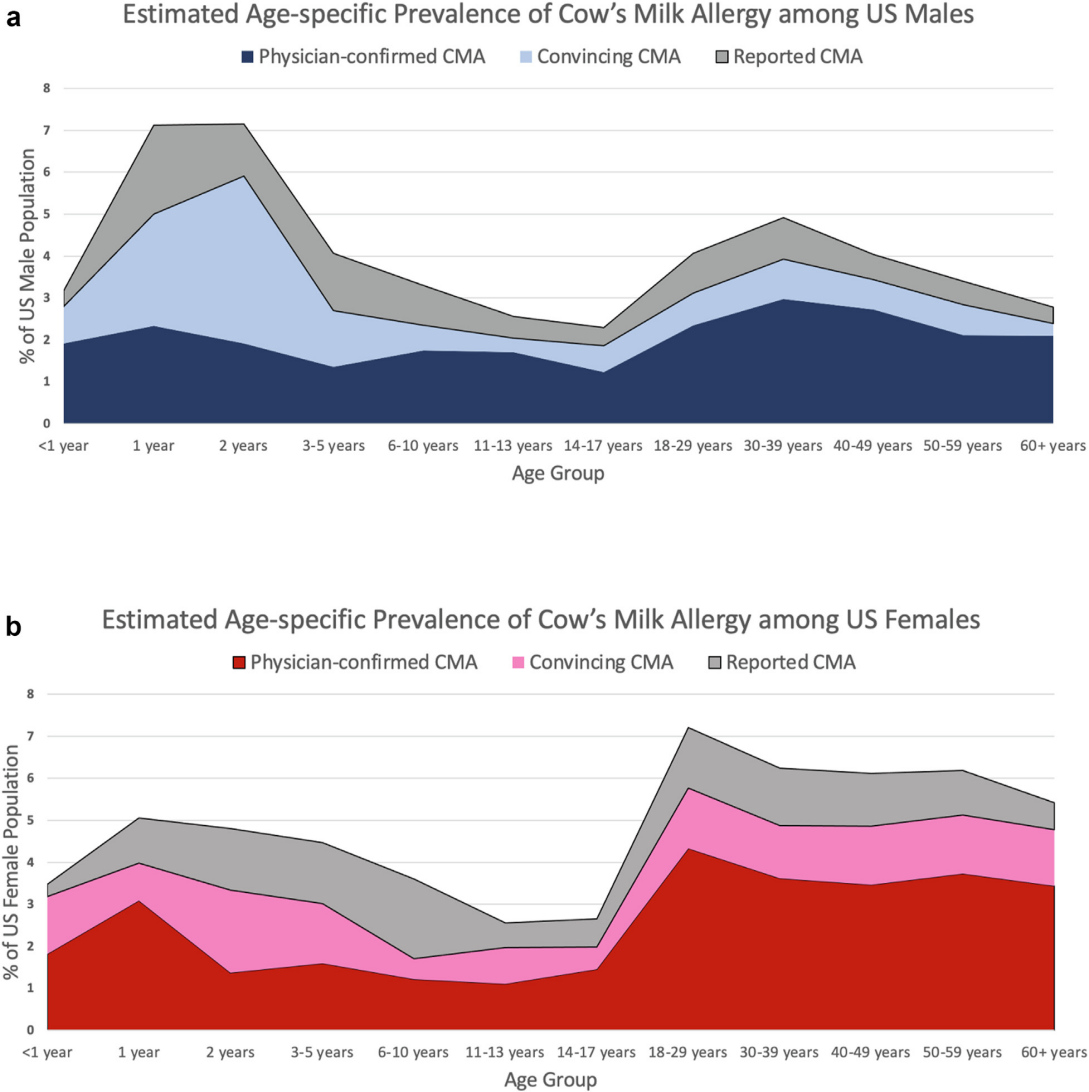
a. Age-specific prevalence of cow's milk allergy among US males. b. Age-specific prevalence of cow's milk allergy among US females

Detailed demographic information for individuals with CMA as well as estimates of CMA prevalence across these demographic strata are provided in Table 1, Table 2, respectively. The prevalence of convincing CMA was elevated among Hispanic [2.5% (95% CI, 2.0–3.0)], non-Hispanic Black [2.3% (95% CI, 1.9–2.8)], and those reporting Multiple and Other races and ethnicities [3.1% (95% CI: 2.2–4.2)] relative to non-Hispanic White [1.6% (95% CI: 1.5–1.8)] and non-Hispanic Asian [1.5% (95% CI: 1.1–2.0)] respondents. CMA was more common among females [2.4% (95% CI: 2.2–2.6)] compared to males [1.4% (95% CI: 1.3–1.6)]. Significant differences were found in CMA prevalence by age, with the highest rates of convincing CMA observed among one-year-olds [3.4% (95% CI: 2.1–5.2)], two-year-olds [4.4% (95% CI: 2.2–8.3)], and three-to-five-year-olds [2.8% (95% CI: 2.1–3.8)]. In contrast, the lowest rates were observed among adolescents aged 11–17 years (1.1%) and adults aged 60 years and older [1.3% (95% CI: 1.1–1.6%)]. These data are visualized in Fig. 1. The estimated prevalence of convincing CMA did not significantly differ across household income strata. However, convincing CMA was significantly more prevalent among individuals with asthma [4.2% (3.6–5.0)], eczema [4.6 (95% CI: 3.9–5.4)], environmental allergies [3.3% (95% CI: 2.9–3.7)] and eosinophilic esophagitis [9.1% (95% CI: 5.2–15.3)]. As reported in Table 3, 72% of individuals with convincing CMA could tolerate baked milk products, whereas 37.3% had additional allergies besides milk—most commonly, peanut (15.1%), shellfish 14.5%), and egg (13.4%).

**Table 2:** Prevalence of reported, convincing, and physician-confirmed cow's milk allergy by weighted sample demographics and comorbid allergic disease.

Variable	Reported milk allergy prevalence % (95% CI)	Convincing milk allergy prevalence % (95% CI)	Physician-confirmed milk allergy prevalence % (95% CI)
Entire sample	**4.7 (4.4–4.9)**	**1.9 (1.8–2.1)**	**0.9 (0.8–1.0)**
Race/ethnicity			
Asian, non-Hispanic	4.7 (3.8–5.7)^b^	1.5 (1.1–2.0)^c^	0.8 (0.5–1.1)^a^
Black, non-Hispanic	4.8 (4.1–5.6)^b^	2.3 (1.9–2.8)^c^	0.9 (0.6–1.3)^a^
White, non-Hispanic	4.3 (4.1–4.6)^b^	1.6 (1.5–1.8)^c^	0.8 (0.7–0.9)^a^
Hispanic	5.3 (4.6–6.1)^b^	2.5 (2.0–3.0)^c^	1.2 (0.9–1.5)^a^
Multiple and other, non-Hispanic	6.3 (5.0–7.8)^b^	3.1 (2.2–4.2)^c^	1.5 (1.0–2.3)^a^
Born in the US			
Yes	4.7 (4.4–4.9)	1.9 (1.8–2.1)	1.0 (0.9–1.1)
No	4.8 (3.9–5.9)	1.7 (1.2–2.2)	0.6 (0.4–1.1)
Sex			
Female	5.6 (5.3–6.0)^c^	2.4 (2.2–2.6)^c^	1.1 (1.0–1.3)^c^
Male	3.6 (3.3–4.0)^c^	1.4 (1.3–1.6)^c^	0.7 (0.6–0.8)^c^
Age			
<1 year	3.4 (2.0–5.6)^c^	1.5 (1.0–2.3)^c^	0.3 (0.2–0.6)^c^
1 year	6.0 (4.3–8.5)^c^	3.4 (2.1–5.2)^c^	1.6 (1.0–2.6)^c^
2 years	6.0 (3.7–9.7)^b^	4.4 (2.2–8.3)^c^	1.3 (0.8–2.2)^c^
3–5 years	4.3 (3.4–5.3)^c^	2.8 (2.1–3.8)^c^	1.4 (1.0–2.0)^c^
6–10 years	3.4 (2.8–4.1)^c^	1.9 (1.5–2.5)^c^	1.4 (1.0–1.9)^c^
11–13 years	2.6 (1.8–3.6)^c^	1.1 (0.8–1.6)^c^	0.5 (0.4–0.8)^c^
14–17 years	2.5 (2.0–3.0)^c^	1.1 (0.9–1.4)^c^	0.6 (0.4–0.7)^c^
18–29 years	5.9 (5.3–6.7)^c^	2.4 (2.0–2.9)^c^	1.2 (1.0–1.6)^c^
30–39 years	5.6 (5.0–6.4)^c^	2.3 (1.9–2.8)^c^	1.2 (0.9–1.5)^c^
40–49 years	5.1 (4.4–5.8)^c^	2.0 (1.6–2.4)^c^	0.9 (0.7–1.2)^c^
50–59 years	4.8 (4.2–5.4)^c^	1.9 (1.6–2.2)^c^	0.8 (0.6–1.1)^c^
60+ years	4.0 (3.6–4.5)^c^	1.3 (1.1–1.6)^c^	0.5 (0.4–0.7)^c^
Household income, $			
<25,000	4.3 (3.8–4.8)	2.0 (1.7–2.3)	0.8 (0.6–1.1)^c^
25,000–49,000	4.9 (4.4–5.4)	2.0 (1.7–2.3)	0.8 (0.8–1.0)^c^
50,000–99,999	5.1 (4.6–5.5)	2.1 (1.9–2.4)	1.3 (1.1–1.5)^c^
100,000–149,000	4.3 (3.7–4.9)	1.7 (1.3–2.1)	0.7 (0.6–1.0)^c^
≥150,000	4.5 (3.7–5.4)	1.6 (1.2–2.2)	0.7 (0.5–1.0)^c^
Other conditions			
Asthma	7.9 (7.1–8.8)^c^	4.2 (3.6–5.0)^c^	2.3 (1.9–2.8)^c^
Eczema	9.5 (8.3–10.8)^c^	4.6 (3.9–5.4)^c^	2.4 (1.9–2.9)^c^
Environmental allergies	7.7 (7.0–8.4)^c^	3.3 (2.9–3.7)^c^	1.7 (1.4–2.0)^c^
EoE	2.9 (8.0–20.1)^c^	9.1 (5.2–15.3)^c^	7.1 (3.7–13.2)^c^
BMI [Mean (95% CI)]	22.8 (22.2–23.4)	21.0 (20.2–21.9)	20.0 (19.1–20.8)

Abbreviations: BMI, body mass index; CI, confidence interval; EoE, eosinophilic esophagitis.

a p<.05.

b p < .01.

c p < .001

**Table 3:** Comorbid food allergies of individuals with convincing milk allergy.

Variable	Among individuals with convincing milk allergy % (95% CI)	Among individuals with convincing specific food allergies besides milk % (95% CI)
Baked Milk Tolerance	71.6 (63.3–78.7)	N/A
Have a top 9 food allergy besides milk	37.3 (30.0–45.3)	100
Comorbid food allergies		
Peanut	15.1 (12.9–17.7)	31.3 (29.6–33.0)
Egg	13.4 (10.8–16.6)	13.7 (12.3–15.2)
Shellfish	14.5 (12.3–17.0)	42.6 (40.6–44.6)
Tree nuts	9.3 (7.5–11.6)	19.8 (18.4–21.2)
Wheat	11.4 (9.2–13.9)	12.1 (10.8–13.5)
Soy	10.1 (8.1–12.5)	9.8 (8.7–11.1)
Fin fish	6.6 (5.2–8.3)	13.3 (12.1–14.7)
Sesame	2.4 (1.8–3.3)	3.8 (3.2–4.6)

Abbreviation: CI, confidence interval

#### Body mass index

On average, body mass indices were lower among individuals with convincing CMA [Mean = 21.0 (95% CI, 20.2–21.9)] than those with allergies to the other top 8 food allergens [Mean = 22.8 (22.3–23.2)] and the general population [Mean = 22.6 (22.5–22.8)]. This difference remained after adjustment for age, sex, race and ethnicity, and asthma status in the multiple regression models irrespective of whether the individuals with convincing CMA were compared to those with allergies to the other top 8 allergens [B = −1.03; (95% CI: −1.70, −0.37] or members of the general population without CMA [B = −0.91; (95% CI: −1.63, −0.18)]. When standardized World Health Organisation (WHO) cutoffs were applied, a greater proportion of individuals with convincing CMA were categorized as underweight (14.0%), compared to both the general population (8.9%) and those with other food allergies besides CMA (8.4%). Fitting 2 additional regression models with a replacement for the indicator for convincing CMA with an indicator for a) convincing CMA with baked milk allergy; or b) convincing CMA with baked milk tolerance, revealed that the association with BMI was much stronger among individuals with baked milk allergy [B = −2.75; (95% CI: −4.33, −1.17)] compared to those with baked milk tolerance [B = −0.36; (95% CI: −1.16, 0.44)].

#### Age-specific onset of CMA

**Fig. 2:**
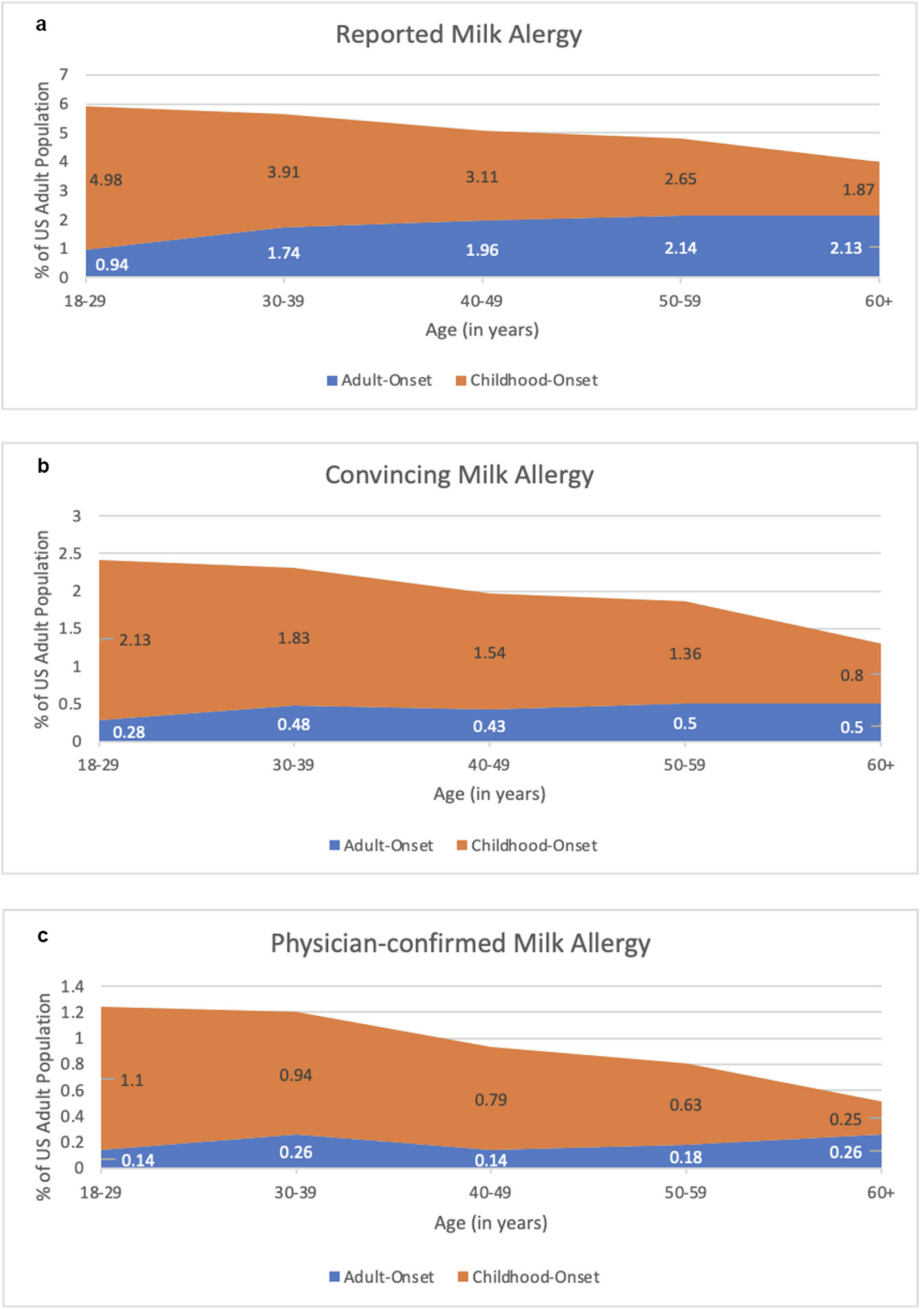
Age-specific prevalence of cow's milk allergy by age at time of first reported milk-allergic reaction (i.e. allergy onset), plotted by case definition utilized. (a: Reported cow's milk allergy; b: Cow's milk allergy with “convincing” IgE-mediated symptomatology; c: Physician-confirmed cow's milk allergy with “convincing” IgE-mediated symptomatology)

The reported onset of CMA was evaluated across all age strata, as shown in Fig. 2. Most cases of convincing CMA had their onset during childhood, but report of convincing adult-onset CMA was increasingly common across older age strata. For example, among 18–19-year-olds only 12% of convincing CMA cases had their onset after age 17, whereas for adults aged 60+, 38% reported that their first allergic reaction to milk occurred after age 17. Notably, report of adult-onset CMA was more common among those reporting an accompanying allergic reaction history that did not meet our stringent “convincing” criteria for IgE-mediated CMA.

#### Outgrown allergy

Among individuals who outgrew their convincing CMA (Table 4), a disproportionately high number—68.5% (60.9%–75.2%)—were of White, non-Hispanic race and ethnicity, while Non-Hispanic Black [7.3% (5.0%–10.7%)], Hispanic [19.4% (13.0%–27.9%)], and those reporting Multiple and Other races and ethnicities [3.4% (2.0%–5.9%)] were under-represented relative to the general US population of individuals with convincing CMA. Similarly, females were less likely than males to outgrow a milk allergy [54.1% (46.0%–62.0%)], as were those with physician-diagnosed eczema/atopic dermatitis [10.8% (7.2%–15.9%)] compared to those without such a diagnosis. Individuals reporting cutaneous symptoms [57.5% (49.6%–65.0%)] were more likely to report outgrowing their CMA, whereas individuals reporting gastrointestinal [34.9% (28.3%–42.1%)] and/or cardiovascular [14.2 (9.8%–20.1%)] symptoms were less likely to report outgrowing their CMA than were patients who did not report those specific symptoms during their most severe allergic reaction to cow's milk.

**Table 4:** Outgrown milk allergy.

Variable	% of each US subpopulation who outgrew a convincing milk allergy^a^ Row % (95% CI)	Distribution of demographic and clinical characteristics among individuals who outgrew a convincing milk allergy^b^ Column % (95% CI)	Difference in estimated frequency of demographic and clinical characteristics between individuals who outgrew a convincing milk allergy - individuals with current convincing milk allergy^c^ Δ	Survey-adjusted Wald F Test; two-tailed P value
Age				
<1 year	0.1 (0.1–0.3)	0.3 (0.1–0.6)	−0.6	F = 2.0; p = .03
1 year	1.1 (0.3–3.7)	2.0 (0.6–6.6)	+0.1	
2 years	0.3 (0.1–0.7)	0.6 (0.2–1.5)	−2.3	
3–5 years	0.8 (0.4–1.4)	4.6 (2.6–8.1)	−0.7	
6–10 years	0.7 (0.5–1.0)	7.1 (4.8–10.3)	+0.8	
11–13 years	0.7 (0.4–1.4)	4.6 (2.5–8.4)	+2.4	
14–17 years	0.6 (0.4–1.0)	5.6 (3.5–8.9)	+2.5	
18–29 years	0.6 (0.4–0.8)	15.8 (11.3–21.8)	−4.9	
30–39 years	0.9 (0.6–1.4)	20.3 (14.1–28.3)	+4.5	
40–49 years	0.5 (0.3–0.7)	9.8 (6.2–15.0)	−3.4	
50–59 years	0.5 (0.3–0.7)	10.9 (7.2–16.0)	−2.7	
60+ years	0.5 (0.4–0.7)	18.5 (13.6–24.7)	+4.4	
Has multiple current food allergies	2.6 (1.9–3.5)	62.0 (51.8–71.3)	+5.6	F = 1.1; p = .29
Race and ethnicity				
Asian, non-Hispanic	0.2 (0.1–0.5)	1.4 (0.6–3.1)	−1.5	F = 5.3; p = .002
Black, non-Hispanic	0.4 (0.3–0.5)	7.3 (5.0–10.7)	−7.0	
White, non-Hispanic	0.7 (0.6–0.8)	68.5 (60.9–75.2)	+15.6	
Hispanic	0.7 (0.4–1.1)	19.4 (13.0–27.9)	−3.1	
Multiple and other	0. (0.3–0.8)	3.4 (2.0–5.9)	−4.0	
Sex				
Female	0.6 (0.5–0.8)	54.1 (46.0–62.0)	−9.6	F = 4.6; p = .03
Male	0.6 (0.4–0.7)	45.9 (38.1–54.0)	+9.6	
Other conditions				
Asthma	1.3 (1.0–1.8)	26.2 (20.3–33.1)	−0.8	F = 0.04; p = .84
Eczema	1.0 (0.7–1.5)	10.8 (7.2–15.9)	−4.9	F = 3.1; p = .08
Environmental allergies	1.3 (1.0–1.6)	41.8 (34.3–49.7)	+8.7	F = 4.3; p = .04
EoE	1.2 (0.4–3.6)	0.3 (0.1–1.0)	−0.5	F = 2.0; p = .16
Organ system involvement during most severe milk-allergic reaction				
Skin/mucosal tissue	30.4 (25.5–35.8)	57.5 (49.6–65.0)	+18.3	F = 13.3; p = .0003
Gastrointestinal	19.9 (16.2–24.2)	34.9 (28.3–42.1)	−9.8	F = 4.9; p = .03
Respiratory	24.8 (18.7–32.1)	30.4 (23.5–38.2)	+2.1	F = 0.11; p = .73
Cardiovascular	16.4 (11.5–22.7)	14.2 (9.8–20.1)	−6.4	F = 6.4; p = .01

Abbreviation: CI, confidence internal; EoE, eosinophilic esophagitis.

Notes regarding interpretation of presented column values:

a These values reflect the raw % of individuals in each subpopulation who report outgrowing a convincing milk allergy. For example, 0.4% of Non-Hispanic Black Americans have outgrown a convincing milk allergy compared to 0.7% of Non-Hispanic White Americans.

b These values reflect the relative distribution of each characteristic used to define the above subpopulations among individuals who report outgrowing a convincing milk allergy. For example: among US individuals who have outgrown a convincing milk allergy, 54.1% are female and 26.2% have asthma.

c These values reflect the raw difference in the relative distribution of each characteristic between individuals who report outgrowing a convincing milk allergy vs. individuals with a current convincing milk allergy (i.e. the values reported in Table 1. For example: among individuals who have outgrown a convincing milk allergy, Non-Hispanic White individuals are over-represented (by 15.6 percentage points) compared to the % of non-Hispanic White individuals with a current convincing milk allergy

#### Severe reaction symptoms

Supplementary Table 1 shows the symptoms reported during the more severe allergic reactions to milk amongst those with convincing CMA overall, along with those with and without baked milk tolerance. Symptoms are divided into organ systems, which are then further categorized into specific symptoms within those systems.

Gastrointestinal symptoms were the most commonly reported symptoms of all organ systems, present in 44.1% (95% CI, 40.3%–48.0%) of those with a convincing milk allergy, with a comparable frequency between those with and without baked milk tolerance. Amongst the gastrointestinal symptoms, diarrhea [59.8% (95% CI, 56.2%–63.3%)] and belly pain [57.8% (95% CI, 54.1%–61.3%)] were most prevalent, with 44.1% (95% CI, 40.3%–48.0%) reporting a history of cow's milk-induced vomiting.

There was a high prevalence of skin/oral/mucosal tissue symptoms, present in 41.4% (95% CI, 37.8%–45.1%) of those with a convincing milk allergy; however, this was more common in those without baked milk tolerance than those with tolerance, 46.8% (95% CI, 39.3%–54.5%) vs 39.2% (95% CI, 35.1%–43.4%), respectively. Amongst the skin symptoms, itching [31.9% (95% CI, 28.7%–35.3%)] and hives [31.7% (95% CI, 28.3%–35.3%)] were most prevalent.

Respiratory symptoms were seen in 29.0% (95% CI, 25.8%–32.3%) of those with a convincing milk allergy, with a greater prevalence amongst those without baked milk tolerance than those with baked milk tolerance, 30.9% (95% CI, 24.4%–38.1%) vs 28.3% (95% CI, 24.8–32.1), respectively. Amongst symptoms of the lung organ system, nasal congestion [18.8% (95% CI, 15.8%–22.1%)] was most prevalent.

The least reported symptoms were those related to the cardiovascular system, present in just 22.9% (95% CI, 20.1%–25.9%) of those with a convincing milk allergy. Report of any cow's milk-induced cardiovascular symptoms was more common among those without baked milk tolerance than those with baked milk tolerance, 28.7% (95% CI, 22.8%–35.5%) vs. 20.6% (95% CI, 17.7%–24.0%), respectively.

#### Allergy severity based on milk tolerance

As shown in Supplemental Table 2, a severe allergic reaction history to milk was reported more frequently among those with CMA and without baked milk tolerance, affecting 42.7% (95% CI, 35.5%–50.2%) compared to 33.5% (95% CI, 29.6%–37.7%) of those with CMA and with baked milk tolerance (p = .03). Reporting a current epinephrine auto injector (EAI) prescription was also more common among individuals with CMA without baked milk tolerance [29.6% (95% CI, 23.8%–36.1%)] compared to individuals with CMA with baked milk tolerance [22.5% (95% CI, 19.5%–25.7%); p = .04]. Individuals with CMA and without baked milk tolerance were also more likely to report a history of one or more food allergy-related emergency department visit [55.8% (95% CI, 48.2%–63.1%)] compared to individuals with CMA and baked milk tolerance [43.4% (95% CI, 39.2%–47.6%); p = .005]. Report of having been treated with an EAI for a severe milk-allergic reaction was 18.9% (95% CI, 13.5%–25.8%) among those without baked milk tolerance compared to 10.6% (95% CI, 8.8%–12.8%) among respondents with CMA and baked milk tolerance, which was statistically significant (p = .003). No significant differences were found for physician-confirmed milk allergy, the presence of multiple allergies, or an ED visit in the last 12 months among those with and without baked milk tolerance, although each of these characteristics was less common among individuals with CMA who also reported tolerance to baked milk compared to those without baked milk tolerance.

#### Symptoms in individuals with baked milk tolerance vs intolerance

Supplementary Table 1 lists the specific symptoms reported by survey respondents with CMA, including a comparison of specific reaction symptoms among those who were allergic to baked milk compared to those who could tolerate baked milk. In general, more severe symptoms (ie, those comprising our “stringent” criteria) were reported more often among individuals with CMA and with allergies to baked milk compared to those with baked milk tolerance; for example, difficulty swallowing was seen in 17.3% (95% CI, 14.9%–20.0%) vs 14.7% (95% CI, 12.4%–17.4%), and throat tightening in 14.2% (95% CI, 12.0%–16.9%) vs 11.4% (95% CI, 9.2%–14.1%), respectively. No significant differences were reported for respiratory symptoms among the two groups. Gastrointestinal symptoms were reported with comparable frequency among individuals with CMA and with baked milk allergy [44.1% (95% CI, 40.3%–48.0%)] compared to those with baked milk tolerance [44.7% (95% CI, 40.4%–49.1%)]. Cardiovascular symptoms were reported more frequently among individuals with CMA and with baked milk allergy [28.7% (95% CI, 22.8%–35.5%)] compared to those with baked milk tolerance [20.6% (17.7%–24.0%)].

#### Food allergy independent measure (FAIM) scores

The validated food allergy independent measure (FAIM) questionnaire was used to assess food allergy-related psychosocial burden and quality of life (QoL) among individuals with CMA with and without baked milk allergy. Higher item- and subscale scores were reported by individuals with convincing CMA who reported baked milk allergy compared to those who reported tolerance to baked milk. This was the case for Q1 through Q4 of the questionnaire, each of which assesses the expectation of adverse food allergy-related outcomes, as well as for Q5, which assesses perceived food allergy-related social limitations, and Q6, which assesses the degree of food allergy-related dietary restriction. This pattern of results held when the entire sample of individuals with CMA was analyzed (Table 5), as well as when the sample was restricted to individuals with only a single allergy (CMA) (Supplemental Table 3). Table 6 shows how mean psychosocial burden associated with having CMA increased as more restrictive diagnostic case definitions for CMA were applied.

**Table 5:** Food allergy independent measure (FAIM) scores^a^ among individuals with convincing milk allergy—a comparison of those with and without reported baked milk tolerance.

	Individuals with CMA and reported baked milk tolerance [Mean (95% CI)] *[Median (IQR)]*	Individuals with CMA and reported baked milk allergy [Mean (95% CI)] *[Median (IQR)]*	Survey-adjusted Wald F Test; two-tailed P value
EO Subscale: *“How big do you think the chance is that you/your child …*	2.9 (2.8–3.0) *2.5 (1.5)*	3.3 (3.1–3.5) *3.25 (1.5)*	F = 18.4; p < .001
Q1: *… will accidentally eat something to which you/they are allergic?*	3.4 (3.3–3.5) *3 (2.0)*	3.7 (3.4–4.0) *4.0 (3.0)*	F = 3.8; p = .05
Q2: … *will have a severe reaction if you/they accidentally eat something to which you/they are allergic?*	3.4 (3.3–3.5) *3.0 (2.0)*	3.9 (3.6–4.1) *4.0 (2.0)*	F = 12.0; p < .001
Q3: *… will die if you/your child accidentally eat something to which you/your child are allergic?*	2.2 (2.1–2.3) *2.0 (1.0)*	2.6 (2.3–2.8) *2.0 (2.0)*	F = 6.0; p = .01
Q4: *… cannot do the right things (or have the right things done by others) should you/your child accidentally eat something to which you/they are allergic?*	2.5 (2.4–2.6) *2.0 (2.0)*	3.0 (2.8–3.3) *3.0 (2.0)*	F = 16.3; p < .001
IM Subscale	3.0 (2.9–3.1) *3.0 (2.5)*	3.9 (3.7–4.2) *4.0 (2.0)*	F = 54.3; p < .001
Q5: *How many products are you/your child unable to eat because of your/their food allergy?*	3.3 (3.2–3.4) *3.0 (3.0)*	4.1 (3.9–4.3) *4.0 (2.0)*	F = 38.4; p < .001
Q6: *How much does your food allergy affect the things you/your child do/does with others?*	2.8 (2.6–2.9) *3.0 (2.0)*	3.7 (3.4–4.0) *4.0 (2.0)*	F = 34.4; p < .001

Abbreviations: CI, confidence interval; CMA, cow's milk allergy; EO, expectation of outcome; IM, independent measure; Q1-Q6, questions 1 thru 6 on the FAIM questionnaire.

a FAIM scores range from 1 to 7

**Table 6:** Food allergy independent measure (FAIM) scores^a^ among patients with reported, convincing, and physician-diagnosed milk allergy.

	Reported milk allergy [Mean (95% CI)] *[Median (IQR)]*	Convincing milk allergy [Mean (95% CI)] *[Median (IQR)]*	Physician diagnosed milk allergy [Mean (95% CI)] *[Median (IQR)]*
EO Subscale: *“How big do you think the chance is that you/your child …*	2.6 (2.6–2.7) *2.5 (1.25)*	3.0 (2.9–3.1) *2.75 (1.5)*	3.2 (3.1–3.4) *3.0 (1.75)*
Q1: *… will accidentally eat something to which you/they are allergic?*	3.4 (3.3–3.5) *3.0 (2.0)*	3.5 (3.4–3.6) *3.0 (2.0)*	3.7 (3.5–3.8) *4.0 (3.0)*
Q2: … *will have a severe reaction if you/they accidentally eat something to which you/they are allergic?*	3.1 (3.0–3.1) *3.0 (2.0)*	3.5 (3.4–3.6) *3.0 (3.0)*	3.8 (3.7–3.9) *4.0 (2.0)*
Q3: *… will die if you/your child accidentally eat something to which you/your child are allergic?*	1.9 (1.8–1.9) *1.0 (1.0)*	2.3 (2.2–2.4) *2.0 (2.0)*	2.6 (2.4–2.8) *2.0 (3.0)*
Q4: *… cannot do the right things (or have the right things done by others) should you/your child accidentally eat something to which you/they are allergic?*	2.3 (2.2–2.4) *2.0 (2.0)*	2.7 (2.6–2.8) *2.0 (3.0)*	2.9 (2.7–3.0) *2.0 (2.0)*
IM Subscale	3.0 (2.9–3.0) *3.0 (2.0)*	3.3 (3.2–3.4) *3.0 (2.0)*	3.4 (3.3–3.5) *3.5 (2.0)*
Q5: *How many products are you/your child unable to eat because of your/their food allergy?*	3.3 (3.2–3.4) *3.0 (2.0)*	3.5 (3.4–3.6) *3.0 (3.0)*	3.7 (3.5–3.8) *4.0 (2.0)*
Q6: *How much does your food allergy affect the things you/your child do/does with others?*	2.6 (2.6–2.7) *2.0 (3.0)*	3.0 (2.9–3.2) *3.0 (2.0)*	3.1 (3.0–3.3) *3.0 (2.0)*

Abbreviation: CI, confidence interval; EO, expectation of outcome; IM, independent measure; Q1-Q6, questions 1 thru 6 on the FAIM questionnaire.

a FAIM scores range from 1 to 7

## Discussion

To our knowledge, this is one of the few studies to comprehensively characterize the population burden of CMA on US children and adults, concluding that 1.9% of the US population reported having CMA with convincing IgE-mediated symptoms. However, fewer than half of those individuals—0.9% of the US population—reported obtaining a physician-diagnosis for their IgE-mediated CMA. In contrast, our population-based survey data indicate that nearly 1 in 20 Americans—or 4.7% of the US population— report that they have a CMA; this translates to about 15.5 million people in the US who may be living with a dairy-restricted diet and associated FA-related psychosocial burden, including fear of experiencing potentially fatal anaphylaxis and a restricted social life. Notably, as compared to previously reported US population-based prevalence estimates based on surveys administered in 2009–2010,^14^ the estimates of convincing (1.9% vs 1.6%) and physician-confirmed (1.2% vs 1.0%) pediatric CMA presented here represent slight increases in disease prevalence and are comparable in magnitude to those observed for peanut allergy^10^^,^^15^ over the past decade. In contrast, estimates from a large European meta-analysis of studies published through 2012 reported the prevalence of self-reported CMA at 2.3% (95% CI 2.1%, 2.5%), CMA diagnosed via skin prick testing (SPT) at 0.3% (95% CI 0.03%, 0.6%), CMA diagnosed via serum IgE testing (sIgE) at 4.7% (95% CI 4.2%, 5.1%),^16^ and estimates based on food challenge or convincing reaction history at 1.6% (95%CI: 1.2%–1.9%). Thus, our findings suggest that self- and parent-proxy reported CMA may be more prevalent than previously reported, while prevalence of convincing and physician-confirmed CMA in the US may have increased slightly over the past decade. These observed discrepancies in estimated prevalence of reported vs physician-confirmed 10.13039/100015007CMA are important to address as they suggest that many patients with undiagnosed allergy may lack key management supports (eg, current epinephrine prescriptions and training in its prompt, appropriate use). At the same time, many individuals may incorrectly believe themselves to be allergic to cow's milk and therefore engage in unnecessary allergen avoidance—experiencing concomitant increases in psychosocial burden as a result.

Cow's milk has been part of the human diet for many years and is used in many baked goods, including cakes, breads, muffins, brownies, and cookies. These foods are readily available and chosen as go-to desserts, celebratory foods and snacks in American culture, especially among school-aged children, making them highly desirable and difficult to avoid. Consistent with previous work, we found that report of stringent allergic reaction symptom or severe reactions after consumption of cow's milk were more common among individuals with CMA who cannot tolerate baked milk compared to those with CMA who lack baked milk tolerance. Our data also revealed that milk allergic individuals who cannot tolerate baked milk had greater psychosocial burden—as measured by the FAIM—compared to those with baked milk tolerance. Physicians should be aware of this difference when educating their patients about CMA and consider baked milk challenges for patients as clinically indicated. Proper patient education, confirmatory testing, and appropriate counseling about food avoidance may lead to less food allergy-related burden and a higher quality of life.

Interestingly, these data—which were collected from a large, nationally representative sample—indicate that current CMA prevalence may have a bimodal distribution, peaking initially at age 1–2 years and then once again at age 18–29 years, after which prevalence attenuates. While it has been widely reported that CMA is most prevalent in infants aged less than 2 years, and further described by data from multiple European birth cohorts (ie, EuroPrevall,^17^ Isle of Wight^18^) indicating that while more than the majority of infants with CMA develop tolerance to cow's milk during childhood, the prevalence of CMA in US adults has been largely unreported. Further US-based studies that incorporate clinical confirmation of disease are clearly needed to confirm this apparent spike in CMA prevalence among US young adults and rule out key differential diagnoses.

These data are also consistent with growing evidence that IgE-mediated food allergies may be disproportionately prevalent among individuals identifying as of Non-Hispanic Black race.^19^ These data suggest that one mechanism that may be driving this apparent disparity is the higher reported rates of outgrown food allergy among Non-Hispanic White respondents relative to both Non-Hispanic Black and Hispanic respondents. In light of previous data suggesting racially disparate access to and quality of allergy care,^20^ this raises the possibility that some of the excess burden of convincing milk allergy observed among specific US racial/ethnic minority populations may be attributable to systematic lack of access to follow-up care, including routine monitoring of specific IgE values for evidence of tolerance development and conducting oral food challenges when clinically indicated. On the flip side, it may also be true that tolerance development is truly more frequent and/or rapid among non-Hispanic White patients, relative to their Black counterparts owing to systematic environmental and/or behavioral differences. In any event these findings indicate that efforts to provide more equitable access to confirmatory food allergy testing and quality follow-up care may be warranted.

In this nationally representative sample of individuals with convincing CMA, over 70% reported tolerance to baked milk products, which is comparable to findings from a previous report^21^ in much smaller, selected clinical samples. Based on these data, individuals with CMA who are allergic to baked milk may, on average, have a more severe phenotype, as indicated by a significantly greater likelihood of reporting a severe reaction history and a food allergy-related emergency department visit, as well as nearly twice the likelihood of reporting having treated a previous milk-allergic reaction with an epinephrine auto-injector. However, these data also indicate that individuals with CMA who are allergic to baked milk also are more likely to have multiple food allergies and current prescriptions for an EAI, as well as somewhat greater likelihood of having a physician-confirmed milk allergy, each of which may influence the likelihood that an EAI would be used during an allergic reaction given that those with multiple and/or physician-confirmed allergies and current EAI prescriptions may be more willing, trained, and able to use an EAI for treatment of acute reactions.

Our data also indicate that the psychosocial burden of cow's milk allergy is likely greater among individuals with CMA who also have baked milk allergy as compared to those with baked milk tolerance. Based on responses on the validated FAIM, compared to their baked milk-tolerant counterparts, individuals with CMA who are also baked-milk allergic reported greater perceived risk of 1) accidental allergen exposure; 2) having a severe allergic reaction upon allergen exposure; 3) fatal food-induced anaphylaxis; and 4) inability to effectively treat an allergic reaction if one occurred. Individuals with CMA and baked milk allergy also reported a greater degree of dietary restriction and food allergy-related social limitation than did their peers who were baked milk tolerant. These findings are similar to those observed in a previous study^22^ comparing children with egg allergy and baked egg allergy vs baked egg tolerance and were observed among patients with a single CMA as well as those with additional common food allergies.

Finally, these data are consistent with previous studies which suggest that CMA can have substantial nutritional implications with impacts on growth that may persist through adulthood. For example, previous findings^23^ from a small cohort of 87 young adults with CMA showed that those with CMA were nearly 4 cm shorter on average than a matched set of participants without CMA, however that study found no significant differences by weight or BMI Z-score. Another analysis^24^ of pediatric data from the 2007–2010 National Health and Nutrition Examination Survey found that children with CMA were shorter, weighed less, and had lower BMIs than their age-matched counterparts without CMA. Using survey-reported height and weight, the present data indicate that, indeed, the BMI of individuals with CMA was lower overall relative to not only the general population but also individuals with the other top 8 food allergies. CMA remained a significant predictor of lower BMI in regression analyses adjusting for acknowledged demographic and clinical factors associated with CMA (ie, age, sex, race and ethnicity, comorbid asthma). Furthermore, when BMI was categorized using established WHO guidelines by age and sex into underweight, normal weight, overweight and obese, we also found that individuals with CMA were significantly more likely to be underweight and less likely to be normal weight than their counterparts without milk allergy. These findings highlight the potential need for targeted nutrition counseling and/or dietician referral for individuals with CMA, to ensure adequate nutrition and discuss the potential benefits of emerging milk allergen immunotherapies, which can facilitate re-introduction of previously avoided dairy foods.

## Limitations

There are several limitations to the findings in this study. While surveys can provide a large amount of information from a representative, population-based sample, recall bias can impact the accuracy of the resulting data. Additionally, our data relied exclusively upon parent-proxy and/or self-report, including report of clinical allergy testing and anthropometry. Due to the cross-sectional design and very large sample recruited for our study, no clinical follow-up was conducted; however, future work should consider doing so, at least for a subset of participants who report convincing IgE-mediated allergy. It is notable that, while not all the respondents categorized as having a “convincing milk allergy” also reported a physician-diagnosis, this was deliberate since estimation of the population-level burden of CMA requires identification of likely IgE-mediated CMA cases, irrespective of whether or not the respondent sought or received clinical confirmation of their reported CMA. Finally, we must acknowledge that these data were collected in 2015–2016—however owing to the relative stability of environmental and other influences on CMA prevalence, severity, and management during these last 6 years, we believe they remain highly relevant and applicable to the present US population-level burden of CMA.

## Conclusion

Overall, the findings indicate that the US population-level burden of true CMA is substantial, with nearly 1 in 20 individuals affected to some degree—corresponding to >15 million people. Not only are these individuals affected by CMA, but their reactions lead to severe symptoms, hospital visits, and EAI use. Appropriate, timely diagnosis of CMA and determination of baked milk allergy or tolerance would likely lead to a reduced burden of disease—particularly among individuals who report having a CMA but do not meet clinical criteria for CMA. These data also suggest that over 2 in 3 individuals with CMA have tolerance to baked milk, indicating the potential for less food restriction and better QoL. Future studies should place more emphasis on understanding the allergic experiences of young adults because they are an understudied group for CMA and may have a higher burden of CMA than previously acknowledged.

## Abbreviations

US, United States; CMA, Cow's Milk Allergy; QoL, Quality of Life; IgE, Immunoglobulin E; IRB, Institutional Review Board; FAIM, Food Allergy Independent Measure; BMI, Body Mass Index; CFI, Confirmatory Fit Index; RMSEA, Root Mean Square Error of Approximation; CI, Confidence Interval; EAI, Epinephrine Auto-Injector; ED, Emergency Department; FA, Food Allergy.

## Funding source

Funding was provided by the National Institute for Allergy and Infectious Disease R21AI135702.

This information or content and conclusions are those of the authors and should not be construed as the official position or policy of, nor should any endorsements be inferred by the NIAID or the U.S. Government. The funding agencies had no involvement in the collection, analysis, or interpretation of data; in the writing of the report; or in the decision to submit the article for publication.

## Availability of data and materials

The data will be made available on reasonable request to the corresponding author and conditional on Institutional Review Board approval.

## Author contributions

CMW made substantial contributions to the conception and design of the study, acquisition of data, analysis and interpretation of data as well as to revision of the article and approved the submitted version. AA and DG made substantial contributions to the interpretation of data as well as prepared drafts of and revisions to the article and approved the submitted version. RSG made substantial contributions to the conception and design of the study, acquisition of data, interpretation of data as well as to revision of the article and approved the submitted version.

## Ethics statement

This study was approved by the Institutional Review Boards of the nonpartisan and objective research organization NORC at the University of Chicago and Northwestern University. Either written or oral informed consent was obtained from all study participants. The Northwestern University IRB number is: STU00202279

The authors take collective responsibility for this work. They attest that this manuscript provides an accurate account of the work performed and an objective discussion of its significance.

## Declaration of competing interests

Dr. Warren reports research support from the National Institute of Allergy and Infectious Disease, Food Allergy Research and Education, and the Sunshine Charitable Foundation.

Dr Gupta reports research support from The National Institute of Health (NIH) (R21 ID # AI135705, R01 ID# AI130348, U01 ID # AI138907), Food Allergy Research Education (FARE), Melchiorre Family Foundation, Sunshine Charitable Foundation, Walder Foundation, UnitedHealth Group, Thermo Fisher Scientific, and Genentech. She has served as a medical consultant/advisor for Genentech, Novartis, and Food Allergy Research & Education in the past year. The remaining authors have nothing to disclose.

## Submission declaration

The authors declare that this manuscript is the authors’ original work and that it has not received prior publication nor is it under consideration for publication elsewhere. All authors listed here have significantly contributed to the work and attest to the validity and legitimacy of the data and its interpretation and agree to its submission to this journal.
