# Supplementary material for: The US population-level burden of cow's milk allergy
Source: World Allergy Organ J. 2022 Apr 21;15(4):100644. doi: 10.1016/j.waojou.2022.100644 (PMC9046619; doi:10.1016/j.waojou.2022.100644)
Supplement: Multimedia component 1 [file mmc1.docx]

**SUPPLEMENTAL MATERIAL**

**Supplemental Tables**

| **Supplemental Table 1.** Symptoms Reported during Most Severe Allergic Reaction to Milk among Respondents with Convincing Cow’s Milk Allergy | | | |
| --- | --- | --- | --- |
|  | Reaction Symptoms to milk among respondents with convincing milk allergy  % (95% CI) | Reaction symptoms to milk among respondents with milk allergy and baked milk tolerance  % (95% CI) | Reaction symptoms to egg among respondents with egg allergy and without baked milk tolerance  % (95% CI) |
| Any Stringent Skin/Oral/Mucosal Tissue Symptom(s) | 41.4 (37.8-45.1) | 39.2 (35.1-43.4) | 46.8 (39.3-54.5) |
| *Hives* | 31.7 (28.3-35.3) | 30.8 (27.0-34.9) | 33.6 (26.7-41.2) |
| Itching | 31.9 (28.7-35.3) | 30.7 (27.0-34.7) | 34.5 (28.4-41.1) |
| Rash | 1.5 (1.0-2.3) | 1.4 (0.8-2.3) | 1.8 (1.0-3.5) |
| *Swelling* | 1.0 (0.5-2.0) | 1.0 (0.4-2.3) | 1.2 (0.4-3.8) |
| Lip/tongue swelling | 13.9 (11.8-16.5) | 13.3 (11.0-15.9) | 15.7 (11.0-22.0) |
| *Difficulty swallowing* | 17.3 (14.9-20.0) | 14.7 (12.4-17.4) | 24.0 (18.2-31.0) |
| Hoarse voice | 8.6 (7.0-10.5) | 8.0 (6.3-10.2) | 10.1 (6.9-14.6) |
| Itchy mouth | 13.7 (11.7-16.1) | 13.6 (11.3-16.3) | 14.1 (10.3-19.1) |
| *Throat tightening* | 14.2 (12.0-16.9) | 11.4 (9.2-14.1) | 21.6 (16.2-28.3) |
| Mouth or throat tingling | 10.3 (7.9-13.4) | 9.5 (6.7-13.3) | 12.6 (8.5-18.2) |
| Any Stringent Respiratory Symptom(s) | 29.0 (25.8-32.3) | 28.3 (24.8-32.1) | 30.9 (24.4-38.1) |
| Chest tightening | 11.5 (9.3-14.1) | 10.4 (8.1-13.1) | 14.4 (9.8-20.8) |
| Nasal congestion | 18.8 (15.8-22.1) | 18.0 (14.5-22.0) | 20.5 (15.3-27.0) |
| Repetitive cough | 11.9 (9.8-14.3) | 10.9 (8.8-13.5) | 14.4 (9.8-20.5) |
| Trouble breathing | 13.2 (10.9-15.9) | 12.6 (10.2-15.4) | 14.9 (10.0-21.6) |
| Wheezing | 14.3 (12.0-17.0) | 14.2 (11.6-17.2) | 14.7 (10.2-20.8) |
| Any Stringent Gastrointestinal Symptom(s) | 44.1 (40.3-48.0) | 44.7 (40.4-49.1) | 42.2 (34.8-50.1) |
| Belly pain | 57.8 (54.1-61.3) | 56.5 (52.3-60.6) | 60.9 (53.7-67.7) |
| Cramps | 53.7 (50.0-57.3) | 51.8 (47.5-56.1) | 58.2 (50.8-65.2) |
| Diarrhea | 59.8 (56.2-63.3) | 58.4 (54.2-62.4) | 63.3 (56.4-69.7) |
| Nausea | 42.4 (38.6-46.2) | 42.0 (37.7-46.3) | 43.6 (36.1-51.3) |
| *Vomiting* | 44.1 (40.3-48.0) | 44.7 (40.4-49.1) | 42.2 (34.8-50.1) |
| Any Stringent Cardiovascular Symptom(s) | 22.9 (20.1-25.9) | 20.6 (17.7-24.0) | 28.7 (22.8-35.5) |
| Chest pain | 5.4 (4.1-7.0) | 5.0 (3.6-7.1) | 6.2 (4.0-9.7) |
| Rapid heart rate | 8.3 (6.7-10.3) | 7.3 (5.6-9.5) | 10.9 (7.4-15.8) |
| Fainting, dizziness, or feeling light headed | 9.9 (8.0-12.2) | 8.4 (6.5-10.8) | 13.8 (9.5-19.5) |
| Low blood pressure | 3.8 (2.6-5.4) | 3.5 (2.2-5.4) | 4.6 (2.4-8.4) |

**Supplemental Table 2.** Milk Allergy Severity amongst those with and without Baked Milk Tolerance

| Milk allergy characteristics | Milk-allergic patients with baked milk allergy  (95% CI) | Milk-allergic patients with baked milk tolerance  (95% CI) | P-value |
| --- | --- | --- | --- |
| Severe reaction to milk | 42.7 (35.5-50.2) | 33.5 (29.6-37.7) | .03 |
| Physician-confirmed Milk Allergy | 53.7 (46.0-61.3) | 46.0 (41.9-50.2) | .09 |
| Multiple Allergies | 62.0 (54.0-69.3) | 54.1 (49.9-58.3) | .08 |
| Current EAI prescription | 29.6 (23.8-36.1) | 22.5 (19.5-25.7) | .04 |
| Lifetime ED Visit | 55.8 (48.2-63.1) | 43.4 (39.2-47.6) | .005 |
| Last 12 months ED Visit | 18.0 (12.3-25.6) | 14.2 (11.3-17.6) | .28 |
| Has treated milk-allergic reaction with EAI | 18.9 (13.5-25.8) | 10.6 (8.8-12.8) | .003 |

Abbreviations: CI, confidence interval; ED, emergency department; EAI, epinephrine auto injector

**Supplemental Table 3**

| Food Allergy Independent Measure (FAIM) Scores^a^ among Patients with Convincing Milk Allergy—comparing patients with and without reported baked milk tolerance (only respondents with only milk allergy and no other top 9 food allergies) | | |
| --- | --- | --- |
|  | Milk allergic patients with reported baked milk tolerance [Mean (95% CI)] | Milk allergic patients with reported baked milk allergy [Mean (95% CI)] |
| EO Subscale:  *“How big do you think the chance is that you/your child…* | 2.6 (2.5-2.7) | 2.8 (2.6-3.0) |
| Q1: *…will accidentally eat something to which you/they are allergic?* | 3.3 (3.2-3.5) | 3.6 (3.1-4.1) |
| Q2: …*will have a severe reaction if you/they accidentally eat something to which you/they are allergic?* | 3.1 (3.0-3.3) | 3.3 (2.9-3.6) |
| Q3: *…will die if you/your child accidentally eat something to which you/your child are allergic?* | 1.8 (1.7-1.9) | 1.9 (1.6-2.1) |
| Q4: *…cannot do the right things (or have the right things done by others) should you/your child accidentally eat something to which you/they are allergic?* | 2.1 (2.0-2.2) | 2.5 (2.2-2.8) |
| IM Subscale | 2.6 (2.5-2.8) | 3.9 (3.4-4.4) |
| Q5: *How many products are you/your child unable to eat because of your/their food allergy?* | 3.0 (2.8-3.1) | 4.1 (3.6-4.6) |
| Q6: *How much does your food allergy affect the things you/your child do/does with others?* | 2.3 (2.2-2.5) | 3.6 (2.9-4.2) |

^a^FAIM scores range from 1-7

Abbreviations: CI, confidence interval; EO, expectation of outcome; IM, independent measure; Q1-Q6, questions 1 thru 6 on the FAIM questionnaire.

**Supplemental Figures**

**Supplemental Figure 1.** “STRINGENT” SYMPTOM LIST


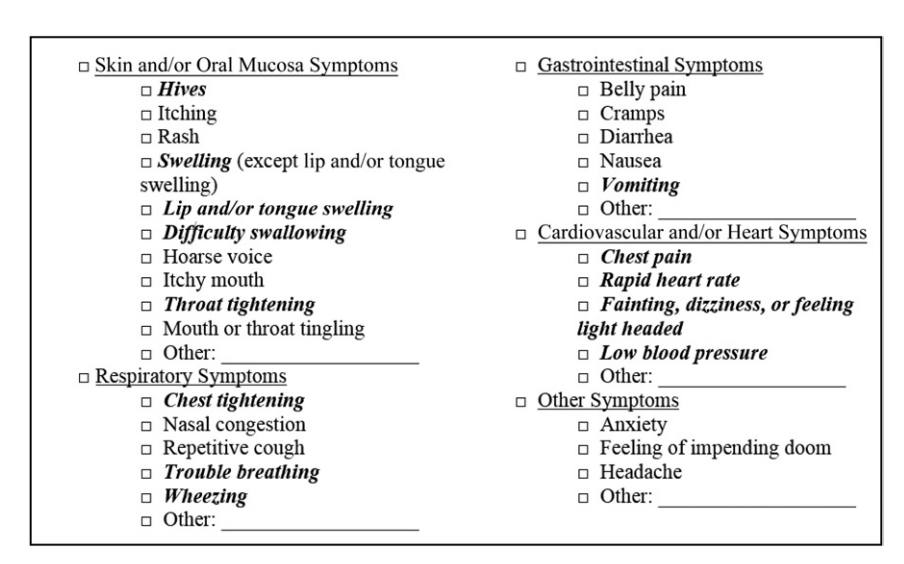


**Supplemental Figure 1 Legend.**

List of allergic reaction symptoms highlighting stringent symptoms indicative of convincingly IgE-mediated food allergy. All symptoms listed were offered as response options in the survey. Symptoms ***italicized in bold*** comprised our expert panel’s stringent symptom list. A “convincing” food allergy required report of at least 1 stringent symptom during a child’s most severe reaction to a given food. A severe reaction consisted of report of at least 2 stringent symptoms from 2 different body systems during the individual’s most severe reaction to a given food.

**Supplemental Figure 2.**

**
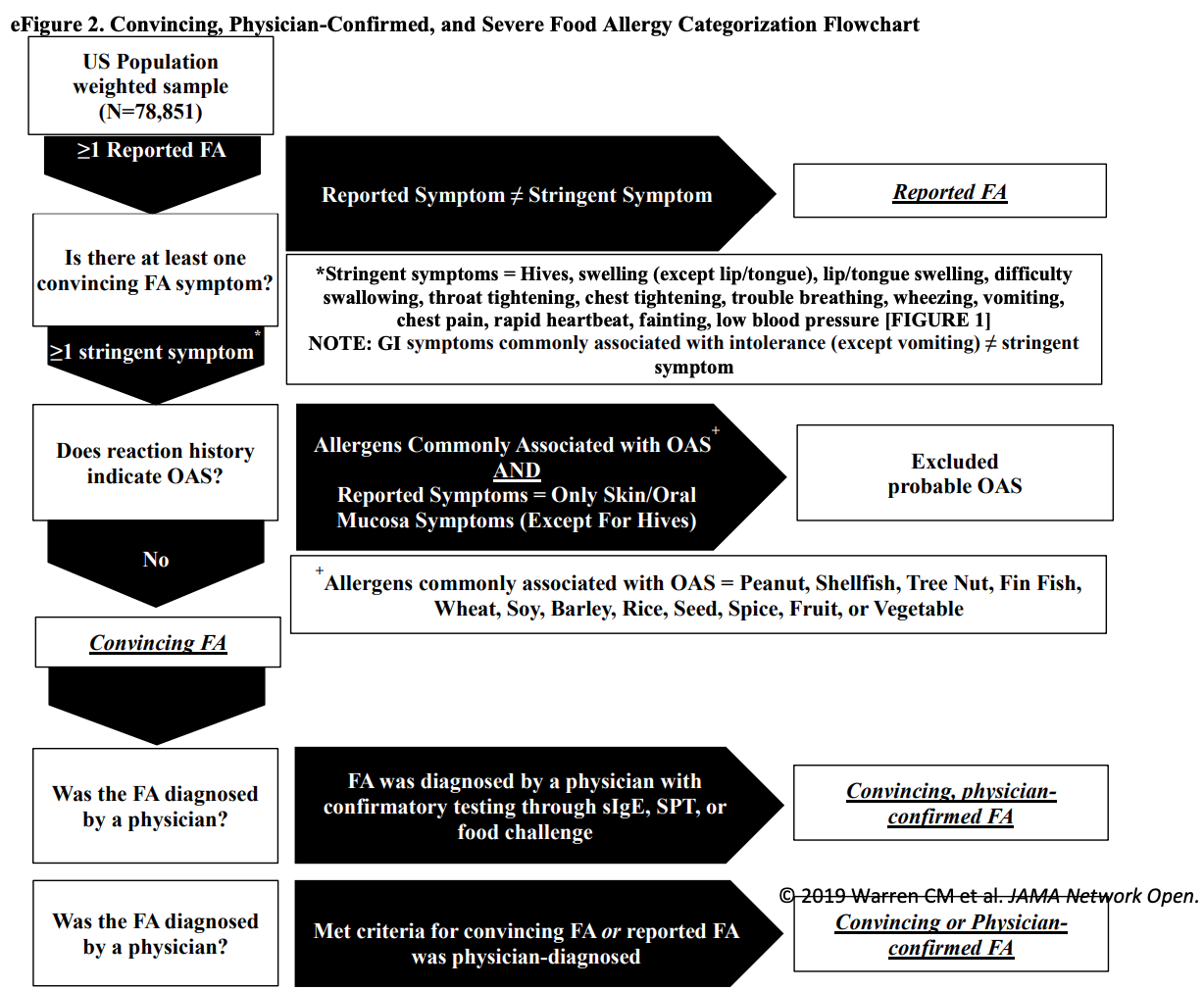
**

**Supplemental Figure 2 Legend:** This flow-chart diagram summarizes how each specific food allergy, including cow’s milk allergy, was defined as “Reported” “Convincing”, or “Physician-confirmed”
